# Enhancing robustness of coupled networks under targeted recoveries

**DOI:** 10.1038/srep08439

**Published:** 2015-02-13

**Authors:** Maoguo Gong, Lijia Ma, Qing Cai, Licheng Jiao

**Affiliations:** 1 Key Lab of Intelligent Perception and Image Understanding of Ministry of Education, International Research Center for Intelligent Perception and Computation, Xidian University, Xi'an, Shaanxi Province 710071, China

## Abstract

Coupled networks are extremely fragile because a node failure of a network would trigger a cascade of failures on the entire system. Existing studies mainly focused on the cascading failures and the robustness of coupled networks when the networks suffer from attacks. In reality, it is necessary to recover the damaged networks, and there are cascading failures in recovery processes. In this study, firstly, we analyze the cascading failures of coupled networks during recoveries. Then, a recovery robustness index is presented for evaluating the resilience of coupled networks to cascading failures in the recovery processes. Finally, we propose a technique aiming at protecting several influential nodes for enhancing robustness of coupled networks under the recoveries, and adopt six strategies based on the potential knowledge of network centrality to find the influential nodes. Experiments on three coupling networks demonstrate that with a small number of influential nodes protected, the robustness of coupled networks under the recoveries can be greatly enhanced.

With the rapid development of electronic, engineering, information and network technologies, modern systems have become more and more complex^1^,^2^,^3^. Nowadays, many systems show a coupling property. The functionality of a complex system depends on not only itself, but also its coupled systems. For instance, a power system is highly coupled with water, oil and transportation systems. The power system relies on the water system to cool the generator, the oil system to provide the fuel and the transportation system to transmit the power. The water, oil and transportation systems also rely on the power system to provide the power^4^,^5^,^6^,^7^. The coupling property of networks is important to understand the controllability^1^, robustness^8^,^9^, synchronization^10^, transport^11^, spreading^12^,^13^,^14^,^15^, competitive percolation^16^ and cooperative evolution^17^ of complex systems.

The coupling property makes the coupled networks fragile under failures. This is because a failure would trigger cascading failures in the entire coupled networks. More specifically, nodes failures in one system would trigger nodes damages of its coupled systems, and the damages in turn result in further nodes failures on the first system^8^. The processes above recursively occur, which may lead to the complete collapse of coupled systems^5^,^6^,^7^,^8^,^9^,^18^,^19^. In reality, it is inevitable that coupled systems suffer from devastating damages caused by the internal and external factors (e.g., earthquake, tsunami, hurricane, volcanic eruptions, diseases, short-circuits and human destruction). These damages often result in the cascading failures of the economy, water, energy, electric power, electronic circuit and road systems and cause great inconvenience to our daily life. For instance, on 28th September, 2003, a catastrophic blackout in Italy was caused by the cascading failures between the electrical power system and its coupled communication system^5^. Moreover, these damages will impact on many processes of coupled networks, including influence spreading^14^, opinion formation^20^, random walks^21^, species evolution^22^, structural balance^23^ and community division^24^.

Recent years, it has become a common focus for how to improve the robustness of coupled networks^25^,^26^. Many models have been proposed to enhance the resilience of coupled systems when these systems lose effectiveness under attacks. A representative model is proposed by Parshani et al^8^. This model seeks to decrease the degree of coupling among systems by removing a set of coupled links. With this strategy, the cascading failures can be greatly reduced by decoupling 40% nodes, and the robustness of coupled systems under the attacks can be greatly enhanced. However, the functionality of these systems changes during the process because a few coupled links are removed^19^. Schneider et al.^19^ propose a novel model based on the generation of a few autonomous nodes. The autonomous nodes cannot be triggered damaged when their coupled nodes are suffering from damages. With this strategy, the degree of coupling is greatly decreased in coupled systems, and the robustness of coupled networks can be enhanced with the initial functionality undamaged by establishing 10% autonomous nodes. Moreover, Hu et al.^27^ theoretically analyze the effect of structural inter-similarity of coupled networks on the reduction of cascading failures. Inter-similarity represents the tendency of connected nodes in one network to be interdependent of connected nodes in its coupled networks. The theoretical analysis demonstrates that the cascading failures decrease with the increase in structural inter-similarity, and that the robustness of coupled networks can be enhanced by increasing the inter-similarity. In addition, Reis et al.^28^ theoretically analyze the effect of the relation between the internal structure (e.g., inter-connections) of networks and its pattern of inter-network connections on the stability of coupled networks. As is pointed out, the robustness of coupled systems can be enhanced by improving the function of connections of one network to its coupled networks and by maximizing the size of the maximal mutual part across coupling networks. The theoretical analysis in Ref. 28 also indicates that the systems of networks are stable and robust to damages when their inter-connections are provided by hubs and when the degree of convergence of inter-network connections is moderate. Note that, the work in Refs. 8, 19, 27, 28 can effectively reduce the fragility of coupled networks to malicious attacks. However, they do not take into consideration the robustness of coupled networks under their recoveries.

For the systems to recover functionality soon, it is necessary to reconstruct the damaged systems^29^,^30^,^31^,^32^,^33^,^34^,^35^. However, there are also cascading failures in coupled systems during the recovery processes, which would make it difficult to recover the functionality. To enhance the robustness of coupled networks during the recovery processes, four challenges need to be overcome. The first one is that it is unknown for the model of recovery processes. To address it, Majdandzic et al.^35^ propose global recovery processes based on a general phenomenon. Damaged systems (e.g., human brain and the financial network) can be spontaneously recovered after an inactive period of time. Note that, in the real world, many complex systems (e.g., power system and airway system) have little ability to recover their functionality spontaneously. Moreover, it takes a long time and consumes much energy for the spontaneous recovery processes. In this study, we adopt a targeted recovery model in which the nodes with high degree are iteratively recovered. This simple strategy is widely used in practical applications. The second challenge is that there is no mathematical model that can express the cascading failures between coupled networks during the recoveries. The third challenge is how to evaluate the recovery robustness of coupled networks. And the last challenge is how to enhance the recovery robustness of coupled networks. Few studies have ever addressed the last three challenges so far.

In order to address the last three challenges, in this paper, firstly, we propose a damaged coupling model, and analyze the cascading failures between coupled networks in the recovery processes. Secondly, we extend the work in Ref. 19, and propose an index *R_rc_* to evaluate the robustness of coupled networks under the recoveries. Finally, a technique based on the protection of several influential nodes is presented to enhance the robustness of coupled networks under their recoveries. Thus, the influential nodes can work normally when they or their coupled nodes suffer from damages. Moreover, based on the network-specific knowledge, we adopt six strategies to find the influential nodes. Experiments on three coupling networks demonstrate that the recovery robustness can be greatly enhanced by protecting 5% influential nodes.

## Results

To demonstrate the performance of our method in enhancing the robustness of networks under the recoveries, we test our method on the following three damaged coupling networks.

1. **ER-ER coupled system**: Many traditional networks show a random connection property and they are widely modeled as Erdő-Rényi (ER) random graphs. In ER random graphs, two nodes are linked with probability *q*, and the average degree 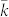 is computed as *N* · *q*, where *N* is the number of nodes^36^. It is important to analyze the robustness of traditional network topology under attacks by studying on coupled ER-ER networks. We test our model on a coupling system between a completely damaged ER network with *N* = 10,000 and 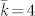 and an ER network with *N* = 10,000 and 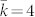.
2. **ER-SF coupled system**: Many modern networks, such as Internet, scientific collaboration, telephone, power grid and airline networks, can be approximated by scale-free (SF) networks with a power-law degree distribution *P*(*u*) = *k*^-*u*^, where *u* is an exponential factor^22^ and *k* represents the nodes degree of networks. Moreover, SF networks and ER networks have different statistic properties and topologies^18^,^37^. Therefore, the analysis of the robustness of coupled ER-SF networks is also of great importance. The experimental ER-SF network is composed of a completely damaged ER network with *N* = 10,000 and 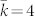 and an SF network with *N* = 10,000 and *u* = 2.5.
3. **Power-SF coupled system**: ER networks have no modularity property with which a few nodes connect densely with each other but link sparsely with the remaining nodes of the network^19^. Besides, many systems show the modularity property. Therefore, it is necessary to analyze the robustness of a coupling network with modularity property. A real U.S. Power Grid network (power) with *N* = 4,941 nodes and *M* = 6,954 edges shows a high modularity property^38^,^39^,^40^,^41^, and its coupled systems, e.g., communication networks, show the scale free property with *u* ranging from 2 to 2.6^42^,^43^. Hence, a coupling system between a completely damaged power network and an SF network with *N* = 4,941 and *u* = 2.2 is analyzed.

All networks are coupled with each other using the model in Refs. 5, 8. This model considers a pair of networks. Each node in one network is randomly coupled with one in the other network.

Table 1 records the robustness *R_rc_* (*λ* = 0.5) of the tested networks under their recoveries. All experimental results are averaged over 50 independent trials, and all algorithms are simulated by MATLAB on a PC with Intel (R), Core (TM), i3 CPU with 3.2 GHZ, 3 GB memory.

The results in Table 1 show that the ER-ER network is robust while the ER-SF and Power-SF networks are fragile to cascading failures under the recoveries. Of the tested networks, the Power-SF network is the most fragile under the recoveries. This is due to two factors. Firstly, the power network has the scale-free property^37^,^43^. The scale-free degree distribution property results in the fact that the Power-SF network is more vulnerable than the ER-ER and the ER-SF networks. Secondly, the power network has the modularity property^40^,^41^ with which the nodes in the same modules are closely linked together. In this case, a node failure may trigger further failures on the nodes of the same module^44^.

We compare six strategies to select the 5% influential nodes: Random, Degree^45^, Betweenness^45^, PageRank^46^, LeaderRank^47^ and Local^48^ choice, for the experimental networks, and the corresponding results are also recorded in Table 1. The results show that the recovery robustness of coupled networks can be greatly enhanced, especially for the Betweenness protection strategy. More specifically, the improvement of recovery robustness can reach 22.65% for the ER-ER network, 114.2% for the ER-SF network and 1,494% for the Power-SF network. Moreover, compared with the random protection, the targeted protections make the ER-ER network more robust to cascading failures during the recoveries. The results also show that the Degree, the PageRank and the LeaderRank protection strategies can get similar results since for the ER and SF networks these three criteria are highly correlated^19^,^49^. It is notable that the Betweenness and the Local protection strategies have similar results in the ER-ER network. However, the Local strategy obtains much smaller *R_rc_* values than the Betweenness strategy in the ER-SF and Power-SF networks. This is because the Betweenness criterion reflects the global nodes information of networks while the Local criterion evaluates the local nodes information. It is in the ER networks rather than the SF and Power networks that the global connections can be reflected by the local information of nodes.

**Figure 1:**
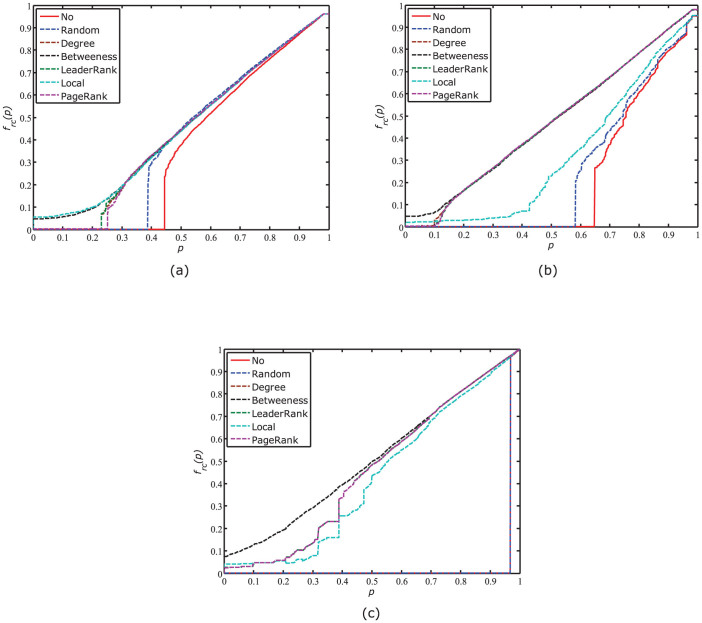
Variations of the fraction of remaining nodes *f_rc_*(*p*) in the largest connected parts with the fraction of recovered nodes *p* for (a) the ER-ER network, (b) the ER-SF network and (c) the Power-SF network. It can be seen that the recovery robustness can be greatly enhanced by protecting 5% influential nodes. We choose the 5% influential nodes in six different ways: Random (blue dotted line), high Degree (brown dotted line), high Betweenness (black dotted line), high LeaderRank (green dotted line), high Local (light green dotted line) and high PageRank (purple dotted line). Results are averaged over 50 independent trials.

In order to further analyze the fragility of experimental networks during the recoveries, we analyze the variations of the remaining fraction of nodes *f_rc_*(*p*) in the largest connected parts with the fraction of recovered nodes *p* in Fig. 1. It shows that without protection strategies, the experimental networks undergo a discontinuous percolation transition where the *f_rc_*(*p*) value abruptly changes from zero to a finite value. More specifically, the ER-ER network, the ER-SF network and the Power-SF network begin to recover their functionality when close to 43%, 63%, and 97% nodes are recovered, respectively. With the protection strategies, especially for the Betweenness protection strategy, the experimental networks begin to recover their functionality when few nodes are recovered. The results in Fig. 1 further demonstrate the effectiveness of the proposed protection strategies on the enhancement of the recovery robustness of coupled networks.

**Figure 2:**
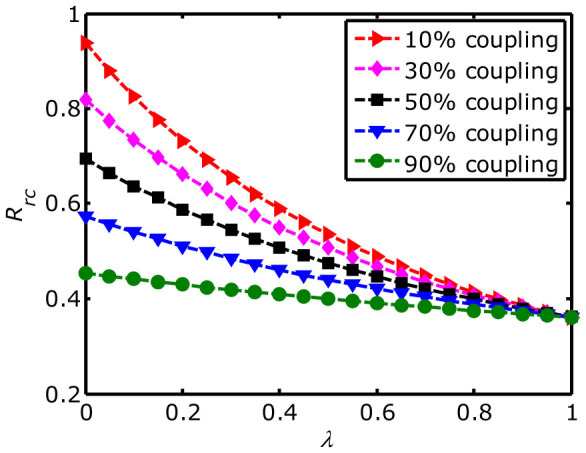
Influences of the parameter *λ* and the coupling degree on the robustness of Power-SF networks. The *R_rc_* values are averaged over 50 independent trials when the *λ* values range from 0.0 to 1 at intervals of 0.05.

Fig. 2 shows the influences of the mixing parameter *λ* and the coupling degree on the recovery robustness of the Power-SF networks. As shown in Fig. 2, *R_rc_* has negative relation with the coupling degree and the parameter *λ*. For a strong coupling network, say 90% coupling, the *R_rc_* value is less sensitive to the parameter *λ*. This is because the fraction of surviving nodes in one network is close to that of the ones in its coupled network. However, for a weak coupling network, say 10% coupling, *R_rc_* has negative relation with *λ*. This is because the cascading failures in a weak coupling network are decreased and the damages on the SF network are smaller than those on the power network during the recoveries.

**Figure 3:**
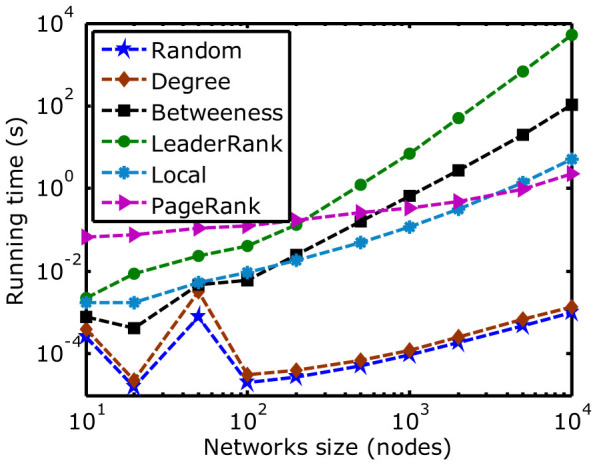
Comparisons of the running time between the adopted protection strategies in ER random networks with different scales.

In order to compare the computational performances of the six protection strategies, we employ them on the ER networks with different scales and record the consumed time in Fig. 3. When the scale of the ER network is small, the six protection strategies can find the influential nodes quickly. When the scale of the ER network is large, it is hard for the LeaderRank and the Betweenness protection strategies to evaluate the importance of nodes in a short period of time. Many systems (e.g., Internet and communication systems) have millions of nodes and links. The LeaderRank and the Betweenness protection techniques cannot tackle it well. Moreover, the Random and Local protection strategies can evaluate the influences of nodes in a short time. However, it is hard for them to improve the recovery robustness of networks by protecting 5% influential nodes, as shown in Table 1. The results in Fig. 3 and Table 1 also show that both the Degree and the PageRank protection strategies can greatly enhance the recovery robustness of large-scale networks in a reasonable time.

## Discussion

The coupling property makes complex systems fragile to cascading failures during the recoveries. How to enhance the robustness of coupled systems with low cost has received many attentions in recent years. In this paper, the cascading failures between coupled networks in the recovery processes are represented by a mathematical model, and the resilience of coupled systems to cascading failures under the recoveries is evaluated by the proposed recovery robustness index. And a protection method for protecting several influential nodes is proposed to enhance the recovery robustness of coupled systems. Experimental results have shown that the recovery robustness of coupled systems is greatly enhanced by the proposed method. Our experiments also demonstrate that the coupled networks with modularity property are more fragile to failures than those with no modularity property. The Degree and the PageRank protection strategies have low computational complexity, and can be used to enhance the robustness of large-scale coupled networks with millions of nodes.

Combined with the spreading process and the robustness of networks, our work can derive a series of interesting issues that are worth further studying. For instance, the studies in Refs. 12,13,14,15 propose the methods for identifying influential spreaders and present the conditions for viral influence spreading. Following these studies, we will extend our work to analyze the effect of the protection of super-influential nodes on the enhancement of the recovery robustness of coupled networks. Moreover, in the real world, the coupling behavior of real systems are more complex than that of the model in Refs. 5, 8 and the Degree and PageRank techniques are difficult to find the influential nodes in these systems^15^. Our work will be extended for different types of coupled networks to identify influential nodes. In addition, the study in Ref. 27 finds the relationship between the inter-similarity and the cascading failures of coupled networks. Following this study, we will increase the inter-similarity by finely tuning the links structures of networks, enhancing the recovery robustness of coupled networks. Finally, the study in Ref. 28 analyzes the relationship between the stability of networks and the internal and external connections of networks. It is worth further studying on the analysis of the relationship between the recovery robustness and the internal and external connections of coupled networks.

## Methods

### Recovery processes

In a system with two coupled subsystems, it is notable that both subsystems are likely to suffer from failures. In practical applications, the probability of damages on both subsystems is smaller than that on one of the subsystems. Moreover, the system cannot operate normally when one of its subsystems suffers from devastating damages. According to those phenomena, a damaged coupling network can be modeled as *G* = (*C*, *D*, *E_CD_*), where *E_CD_* denotes the coupled links between network *C* and *D*. In the model, each node in network *C* is randomly coupled with one node in network *D*, and network *C* suffers from devastating damages. In this case, the network *D* fails to work as well because its coupled network *C* is damaged.

In real-world applications, the damaged systems can be recovered by reconstructing the damaged entities gradually. Reconstructing a damaged system can be regarded as an inverse problem of attacking a system, and it can be modeled as a process in which the damaged nodes in networks are gradually recovered. In the recovery processes, there is an inter-propagation of recoveries in coupled networks. Nodes in network *D* can be triggered to work normally if their coupled nodes in network *C* have been recovered.

### Cascading failures in the recovery processes

**Figure 4:**
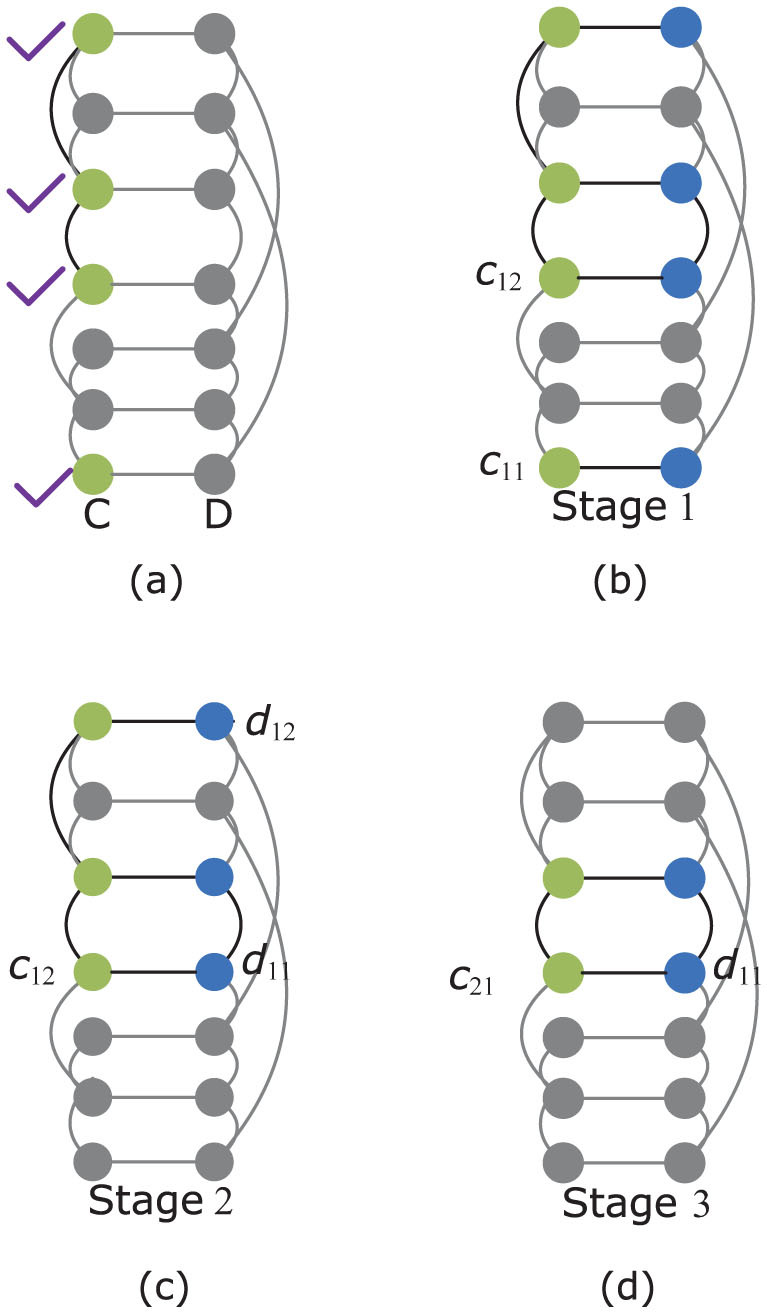
Illustration of a cascade of failures on a toy coupling network with 4 recovered nodes. The purple check marks point out the recovered nodes. Nodes and links painted in gray cannot operate normally. (a) 4 nodes in network *C* are initially recovered. (b) Stage 1: the initial recoveries on network *C* trigger the nodes recoveries of network *D*. (c) Stage 2: the nodes of network *C* that are not in the largest connected part are failed, and the failures trigger the nodes failures of network *D*. (d) Stage 3: the nodes of network *D* that are not in the largest connected part are failed, and the failures in turn result in the nodes failures of network *C*.

There are cascading failures in coupled systems during the recovery processes. Assuming that a fraction of nodes *p* of network *C* has been reconstructed, firstly, the coupled nodes in network *D* are triggered to work normally. Then, cascading failures occur because the recovered nodes in network *C* (*D*) may be scattered in a few unconnected clusters. The recovered nodes in network *C* (*D*) which are not in the largest connected parts would lose their functionality, and the failures in network *C* (*D*) will trigger the failures of the coupled nodes in network *D* (*C*). The above failures recursively occur when there are no further failures in both networks *C* and *D*. A schematic illustration of a cascade of failures on a toy coupling network with 4 recovered nodes of network *C* is given in Fig. 4.

The cascading failures in the recovery process in which a fraction of nodes *p* of network *C* is recovered can be expressed as equation (1)
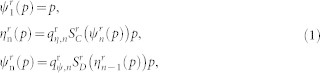
where 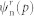 (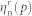) is the fraction of surviving nodes in network *C* (*D*) at the (n-1)-th coupled process when the fraction of nodes *p* in network *C* is initially recovered, 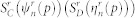 represents the ratio of nodes in the largest connected part of the recovered network *C* (*D*), and 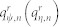 is the fraction of nodes in network *C* (*D*) which is coupled with the recovered nodes in network *D* (*C*). When 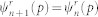 and 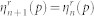, the cascading failures end. In this case, we express 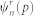 and 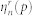 as *ψ^r^*(*p*) and *η^r^*(*p*), respectively.

### Recovery robustness in coupled networks

According to the percolation theory, the functionality of a damaged network is determined by its remaining largest linked part^5^,^8^, and the attack robustness considers the remaining functionality of the network under all possible damages^44^,^50^,^51^. For a single network with *N* nodes, the attack robustness can be calculated as Ref. 51
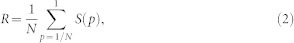
where *S*(*p*) represents the fraction of nodes in the largest linked parts when the fraction of nodes *p* of the network loses the effectiveness. The normalization factor 1/*N* is designed to compare the robustness of networks that are with different scales^51^.

For a coupling network *G* = (*C*, *D*, *E_CD_*), its functionality depends on not only the remaining functionality of network *C*, but also that of network *D*. Therefore, it is necessary for the recovery robustness *R_rc_* of the coupling network to consider the functionality integrity of both networks *C* and *D* during the recoveries. *R_rc_* is computed as
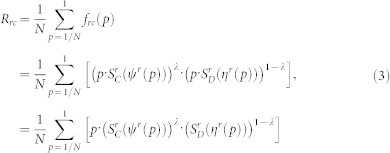
where *f_rc_*(*p*) is the remaining functionality integrity of the coupled network after the fraction of nodes *p* in network *C* has been recovered, and *ψ^r^*(*p*) (*η^r^*(*p*)) is the fraction of nodes in the largest linked parts of network *C* (*D*). The values of *ψ^r^*(*p*) and *η^r^*(*p*) can be computed by equation (1) when 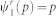. *λ* is a mixing parameter ranging from 0 to 1. When *λ* = 0 or *λ* = 1, the robustness of the coupled network system is determined by that of *D* or *C*, respectively.

The recovery models are mainly divided into two categories: random and targeted. However, it is hard for a random model to recover the functionality of coupled networks with low cost. Therefore, we mainly consider a targeted model in this study. More specifically, the degree-based targeted recovery model widely used in practical applications is adopted.

### Enhancement of the recovery robustness of coupled networks by protecting influential nodes

The functionality of many systems is controlled by a set of influential entities. These systems cannot operate normally once their influential entities are damaged. In real-world applications, complex systems have their own strategies for resisting unpredictable failures. For instance, in medical systems, all medical institutions have backup power generations for providing medical assistance for severe patients. In the coupled bank and computer systems, the influential transaction bank data are protected from hackers' targeted attacks. The common purposes of those strategies are that the influential entities are not to be damaged when they or their coupled entities suffer from damages. Based on the purposes above, we devise a systematic technique aiming at protecting several influential nodes to reinforce the robustness of coupled networks under the recoveries.

The reasons why the proposed strategy can reinforce the robustness of coupled networks under the recoveries are as follows. Firstly, damages on the protected nodes of one network will not lead to the nodes failures of its coupled network. It implies that the degree of coupling between two networks is reduced. According to the results found in Refs. 8, 19, the robustness of coupled networks can be enhanced with the degree of coupling decreasing. Secondly, the protected nodes would not lose their effectiveness when they are damaged. It means that the damages on the networks are decreased, which results in the improvement of the robustness of coupled networks. Moreover, the proposed strategy can reduce the influence caused by cascading failures during the recovery processes. Finally, extensive experiments demonstrate the effectiveness of the proposed strategy on the enhancement of the recovery robustness of coupled networks.

Many criteria based on the network-specific knowledge have been presented for evaluating the influence of nodes^13^. As many real networks have thousands of nodes and links, it is necessary to consider both the computational complexity and the efficiency of those criteria. In this study, we mainly adopt the following well-known criteria to measure the influences of nodes.

**Random** (*I_r_*). In Random centrality, the influence of a node *I_r_*(*i*) is computed as
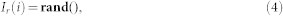
where **rand**() is the function for generating a random value in the range of 0 to 1.

**Degree** (*I_d_*). In Degree centrality, the influence of a node *I_d_*(*i*) is highly related to its degree, and it is computed as Ref. 45
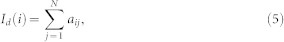
where *a_ij_* represents the connection between nodes *v_i_* and *v_j_*. If there is an edge between nodes *v_i_* and *v_j_*, *a_ij_* = 1, if not *a_ij_* = 0.

**Betweenness** (*I_b_*). In Betweenness centrality, the influence of a node *I_b_*(*i*) is evaluated by the number of the shortest paths that pass through the node^45^.
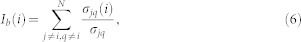
where *σ_jq_* denotes the number of shortest paths from node *v_j_* to node *v_q_*, and *σ_jq_*(*i*) represents the number of shortest paths passing through node *v_i_* from *v_j_* to *v_q_*.

**PageRank** (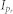). PageRank technique can be described by a random walk process on networks. In PageRank, the influence of a node 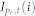 at time *t* in a network is calculated as Refs. 13, 46
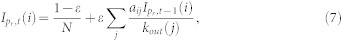
where *ε* is a damping factor for a random walker to move along the links of the network, and it is usually set as a fixed value 0.85^46^. 1-*ε* is the probability for a random walker to jump to a randomly selected node. *k_out_*(*j*) represents the outdegree of node *v_j_*. 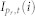 will converge to a stationary value 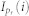 with *t* increased, and the 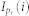 value is the influence of node *v_i_* in the network.

**LeaderRank** (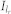). In LeaderRank technique, all nodes, except for the ground node *v_g_*, are initially assigned with one unit of resource. Then, at each state *t*, the resource of each node is evenly distributed to its neighborhoods. This process ends when the resource of each node keeps unchanged, and this state is recorded as *t_c_*. Compared with PageRank, LeaderRank is parameter-free^47^. In LeaderRank centrality, the influence of a node 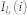 is determined by its resource value at the state *t_c_*, and 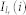 is computed as Ref. 47
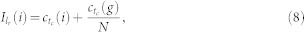
where 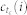 is the resource value of *v_i_* at the state *t_c_*, as computed in equation (9)

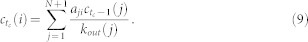


**Local** (*I_l_*). In Local centrality, the influence of a node *I_l_*(*i*) is determined by its nearest and next nearest neighbors, and it is evaluated as Ref. 48
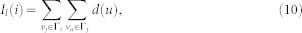
where *d*(*u*) is the number of nodes whose shortest paths from them to node *v_u_* are no more than 2, and Γ*_i_* (Γ*_j_*) is the neighborhood of node *v_i_* (*v_j_*).

## Author Contributions

M.G. and L.M. presented the method and wrote the main manuscript text. L.M. prepared all the figures. Q.C. and L.J. conceived the research. All authors carried out the experimental analysis and reviewed the manuscript.

## Figures and Tables

**Table 1:** Comparisons of the recovery robustness *R_rc_* (*λ* = 0.5) obtained by protecting 5% influential nodes with different strategies

Networks	No	Random	Degree	Betweenness	LeaderRank	Local	PageRank
ER-ER	0.3652	0.4026	0.4319	0.4479	0.4328	**0.4489**	0.4302
ER-SF	0.2220	0.2521	0.4709	**0.4755**	0.4703	0.3242	0.4707
Power-SF	0.0317	0.0320	0.4561	**0.5054**	0.4562	0.4225	0.4560

## References

[b1] Liu Y.-Y., Slotine J.-J. & Barabási A.-L. Controllability of complex networks. Nature 473, 167–173 (2011). 21562557 10.1038/nature10011

[b2] Holme P. & Saramäki J. Temporal networks. Phys. Rep. 519, 97–125 (2012).

[b3] Wu X., Zhu X., Wu G.-Q. & Ding W. Data mining with big data. IEEE Trans. Knowl. Data Eng. 26, 97–107 (2014).

[b4] Liu J. *et al.* Complexity of coupled human and natural systems, Science 317, 1513–1516 (2007). 17872436 10.1126/science.1144004

[b5] Buldyrev S. V., Parshani R., Paul G., Stanley H. E. & Havlin S. Catastrophic cascade of failures in interdependent networks. Nature 464, 1025–1028 (2010). 20393559 10.1038/nature08932

[b6] Cao L., Ou Y. & Yu P. S. Coupled behavior analysis with applications. IEEE Trans. Knowl. Data Eng. 24, 1378–1392 (2012).

[b7] Brummitt C. D., D'Souza R. M. & Leicht E. Suppressing cascades of load in interdependent networks. Proc. Natl. Acad. Sci. USA 109, E680–E689 (2012). 22355144 10.1073/pnas.1110586109 PMC3311366

[b8] Parshani R., Buldyrev S. V. & Havlin S. Interdependent networks: Reducing the coupling strength leads to a change from a first to second order percolation transition. Phys. Rev. Lett. 105, 048701 (2010). 20867893 10.1103/PhysRevLett.105.048701

[b9] Huang X., Gao J., Buldyrev S. V., Havlin S. & Stanley H. E. Robustness of interdependent networks under targeted attack. Phys. Rev. E 83, 065101 (2011). 10.1103/PhysRevE.83.065101 21797429

[b10] Um J., Minnhagen P. & Kim B. J. Synchronization in interdependent networks. Chaos 21, 025106 (2011). 21721784 10.1063/1.3596698

[b11] Morris R. G. & Barthelemy M. Transport on coupled spatial networks. Phys. Rev. Lett. 109, 128703 (2012). 23006001 10.1103/PhysRevLett.109.128703

[b12] Kitsak M. *et al.* Identification of influential spreaders in complex networks. Nat. Phys. 6, 888–893 (2010).

[b13] Pei S. & Makse H. A. Spreading dynamics in complex networks. J. Stat. Mech. 2013, P12002 (2013).

[b14] Hu Y., Havlin S. & Makse H. A. Conditions for viral influence spreading through multiplex correlated social networks. Phys. Rev. X 4, 021031 (2014).

[b15] Pei S., Muchnik L., Andrade Jr J. S., Zheng Z. & Makse H. A. Searching for superspreaders of information in real-world social media. Sci. Rep. 4, 5547 (2014). 24989148 10.1038/srep05547 PMC4080224

[b16] Nagler J., Levina A. & Timme M. Impact of single links in competitive percolation. Nat. Phys. 7, 265–270 (2011).

[b17] Jiang L.-L. & Perc M. Spreading of cooperative behaviour across interdependent groups. Sci. Rep. 3, 2483 (2013). 23963495 10.1038/srep02483 PMC3748424

[b18] Gao J., Buldyrev S. V., Stanley H. E. & Havlin S. Networks formed from interdependent networks. Nat. Phys. 8, 40–48 (2012). 10.1103/PhysRevE.85.066134 23005189

[b19] Schneider C. M., Yazdani N., Aráujo N. A. M., Havlin S. & Herrmann H. J. Towards designing robust coupled networks. Sci. Rep. 3, 1969 (2013). 23752705 10.1038/srep01969 PMC3678138

[b20] Castellano C., Fortunato S. & Loreto V. Statistical physics of social dynamics. Rev. Mod. Phys. 81, 591 (2009).

[b21] Noh J. D. & Rieger H. Random walks on complex networks. Phys. Rev. Lett. 92, 118701 (2004). 15089179 10.1103/PhysRevLett.92.118701

[b22] Scheffer M. & van Nes E. H. Self-organized similarity, the evolutionary emergence of groups of similar species. Proc. Natl. Acad. Sci. USA 103, 6230–6235 (2006). 16585519 10.1073/pnas.0508024103 PMC1458860

[b23] Facchetti G., Iacono G. & Altafini C. Computing global structural balance in large-scale signed social networks. Proc. Natl. Acad. Sci. USA 108, 20953–20958 (2011). 22167802 10.1073/pnas.1109521108 PMC3248482

[b24] Newman M. E. J. Modularity and community structure in networks. Proc. Natl. Acad. Sci. USA. 103, 8577–8582 (2006). 16723398 10.1073/pnas.0601602103 PMC1482622

[b25] Babaei M., Ghassemieh H. & Jalili M. Cascading failure tolerance of modular small-world networks. IEEE Trans. Circuits Syst. II 58, 527–531 (2011).

[b26] Wang J. Robustness of complex networks with the local protection strategy against cascading failures. Safety Sci. 53, 219–225 (2013).

[b27] Hu Y., Zhou D., Zhang R., Han Z. & Havlin S. Percolation of interdependent networks with inter-similarity. Phys. Rev. E 88, 052805 (2013). 10.1103/PhysRevE.88.052805 24329316

[b28] Reis S. D. S. *et al.* Avoiding catastrophic failure in correlated networks of networks. Nat. Phys. 10, 762–767 (2014).

[b29] Ammann P., Jajodia S. & Liu P. Recovery from malicious transactions, IEEE Trans. Knowl. Data Eng. 14, 1167–1185 (2002).

[b30] Kvalbein A., Hansen A. F., Čičic T., Gjessing S. & Lysne O. Multiple routing configurations for fast IP network recovery. IEEE/ACM Trans. Netw. 17, 473–486 (2009).

[b31] Akkaya K., Senel F., Thimmapuram A. & Uludag S. Distributed recovery from network partitioning in movable sensor/actor networks via controlled mobility. IEEE Trans. Comput. 59, 258–271 (2010).

[b32] Chen C.-M., Macwan A. & Rupe J. Network disaster recovery [guest editorial], IEEE Commun. Mag. 49, 26–27 (2011).

[b33] Pocock M. J., Evans D. M. & Memmott J. The robustness and restoration of a network of ecological networks. Science 335, 973–977 (2012). 22363009 10.1126/science.1214915

[b34] Morino K., Tanaka G. & Aihara K. Effcient recovery of dynamic behavior in coupled oscillator networks. Phys. Rev. E 88, 032909 (2013). 10.1103/PhysRevE.88.032909 24125327

[b35] Majdandzic A. *et al.* Spontaneous recovery in dynamical networks. Nat. Phys. 10, 34–38 (2014).

[b36] Erdős P. & Rényi A. On the evolution of random graphs. Magyar Tud. Akad. Mat. Kutató Int. Közl 5, 17–61 (1960).

[b37] Barabási A.-L. & Albert R. Emergence of scaling in random networks, Science 286, 509–512 (1999). 10521342 10.1126/science.286.5439.509

[b38] Watts D. J. & Strogatz S. H. Collective dynamics of ‘small-world’ networks, Nature 393, 440–442 (1998). 9623998 10.1038/30918

[b39] Pahwa S., Scoglio C. & Scala A. Abruptness of cascade failures in power grids. Sci. Rep. 4, 3694 (2014). 24424239 10.1038/srep03694 PMC3892437

[b40] Gong M., Cai Q., Chen X. & Ma L. Complex network clustering by multiobjective discrete particle swarm optimization based on decomposition. IEEE Trans. Evol. Comput. 18, 82–97 (2014).

[b41] Ma L., Gong M., Liu J., Cai Q. & Jiao L. Multi-level learning based memetic algorithm for community detection. Appl. Soft Comput. 19, 121–133 (2014).

[b42] Brandes U. & Erlebach T. Network Analysis: Methodological Foundations. (Springer, Berlin Heidelberg, 2005).

[b43] Nguyen D. T., Shen Y. & Thai M. T. Detecting critical nodes in interdependent power networks for vulnerability assessment. IEEE Trans. Smart Grid 4, 151–159 (2013).

[b44] Ma L., Gong M., Cai Q. & Jiao L. Enhancing community integrity of networks against multilevel targeted attacks. Phys. Rev. E 88, 022810 (2013). 10.1103/PhysRevE.88.022810 24032886

[b45] Freeman L. C. Centrality in social networks conceptual clarification. Social Networks 1, 215–239 (1979).

[b46] Brin S. & Page L. The anatomy of a large-scale hypertextual web search engine. Comput. Netw. ISDN Syst. 30, 107–117 (1998).

[b47] Lü L., Zhang Y.-C., Yeung C. H. & Zhou T. Leaders in social networks, the delicious case. PLoS ONE 6, e21202 (2011). 21738620 10.1371/journal.pone.0021202 PMC3124485

[b48] Chen D., Lü L., Shang M.-S., Zhang Y.-C. & Zhou T. Identifying influential nodes in complex networks. Physica A 391, 1777–1787 (2012).

[b49] Newman M. Networks: An Introduction. (Oxford University Press, New York, 2010).

[b50] Holme P., Kim B. J., Yoon C. N. & Han S. K. Attack vulnerability of complex networks. Phys. Rev. E 65, 056109 (2002). 10.1103/PhysRevE.65.056109 12059649

[b51] Schneider C. M., Moreira A. A., Andrade J. S., Havlin S. & Herrmann H. J. Mitigation of malicious attacks on networks. Proc. Natl. Acad. Sci. USA 108, 3838–3841 (2011). 21368159 10.1073/pnas.1009440108 PMC3053993

